# An Innovative Low-Power, Low-Cost, Multi-Constellation Geodetic-Grade Global Navigation Satellite System Reference Station for the Densification of Permanent Networks: The GREAT Project

**DOI:** 10.3390/s23136032

**Published:** 2023-06-29

**Authors:** Davide Curone, Giovanni Savarese, Mirko Antonini, Raphaël Baucry, Elie Amani, Antonin Boulandet, Marco Cataldo, Paul Chambon, Massimiliano Chersich, Ahmed B. Hussein, Bruno Menuel, Avag Tsaturyan

**Affiliations:** 1 YETITMOVES S.r.l., c/o European Centre for Training and Research in Earthquake Engineering (EUCENTRE), University of Pavia, 27100 Pavia, Italy; mcataldo@yetitmoves.it (M.C.); mchersich@yetitmoves.it (M.C.); 2 SPACEEXE S.r.l., 00131 Roma, Italy; giovanni.savarese@spaceexe.com (G.S.); mirko.antonini@spaceexe.com (M.A.); bakry.hussein@spaceexe.com (A.B.H.); 3 Exagone, Réseau Teria, 94400 Vitry-sur-Seine, France; raphael@reseau-teria.com (R.B.); paul.chambon@reseau-teria.com (P.C.); bruno@reseau-teria.com (B.M.); 4 M3Systems Belgium, 1300 Wavre, Belgium; elie.amani@m3systems.eu (E.A.); antonin.boulandet@m3systems.eu (A.B.); avag.tsaturyan@m3systems.eu (A.T.)

**Keywords:** GNSS, reference station, low cost, multi constellation, Septentrio Mosaic X5, permanent networks densification

## Abstract

Geodetic-grade Global Navigation Satellite System (GNSS) receivers designed to implement permanent stations represent the most complex and costly technology in the field of GNSS instrumentation. On the other hand, a large number of innovative applications, highly demanding in terms of positioning precision and accuracy, is pushing the implementation of networks of permanent stations with a higher and higher spatial density. In this scenario, the development of brand new GNSS reference stations, which combine the most advanced technologies in the field of data availability and integrity together with reduced costs (of instrumentation, installation and management) is becoming of paramount importance. For this reason, in 2019 the EU Agency for the Space Programme (EUSPA) has financed a research project, called “next Generation gnss REference stATion—GREAT”, aimed at developing and demonstrating the potentiality of a brand new GNSS receiver suitable to implement permanent stations. This paper describes the solution developed by the project consortium, composed of four Small or Medium Enterprises (SMEs) based in Italy, France and Belgium, and the preliminary results achieved in the field tests.

## 1. Introduction

Currently, the permanent networks of Global Navigation Satellite System (GNSS) reference stations are complex infrastructures based on specialized instrumentation and costly software tools. Moreover, installing, setting up and maintaining a reference station implies relevant costs that prevent the wide spread of this technology. As a result, most of the public and private GNSS networks are not very dense in the European territory and much less worldwide.

At the same time, a large number of applications, highly demanding in terms of positioning accuracy, would benefit from the availability of real-time differential corrections from reference stations. It is the case of devices or systems based on the well-known real-time-kinematic (RTK) [1] technique or on post-processing kinematic (PPK) positioning [2]. Moreover, the spread of commercial and operational services based on the innovative PPP-RTK concept [3,4] represents an important boost for the realization of dense networks of GNSS reference stations. It is worth noticing that services relying upon GNSS differential corrections include a wide variety of potential customers, ranging from mass market (e.g., for high performance positioning/navigation on new generation smartphones where raw GNSS measurements are available [5,6]) to more specialized application domains (e.g., for GNSS-based real-time structural and geophysical monitoring [7]). Moreover, some new safe sensitive applications such as autonomous driving [8,9] and drones’ navigation [10] need reliable networks of reference stations that shall be redundant in order to guarantee the continuity of service in case of hardware failures of single stations, but that must be also resilient to spoofing and jamming attacks [11], as in cyber-attacks and emergency situations (e.g., earthquakes or natural disasters). 

In the very last few years, some manufactures started delivering multi-constellation multi-frequency GNSS chipsets, which are low-cost with respect to the standards for high-end GNSS instrumentation but with performance allowing millimetric to centimetric level accuracy, respectively, in static-differential and RTK, therefore similar to the state-of-the art professional GNSS receivers. Besides very low-cost chipsets mostly suitable for mass market applications (as in the case of Broadcom BCM47755 [12]), chipsets such as U-Blox ZED-F9P [13,14] or Septentrio Mosaic X5 [15] have demonstrated impressive performances with a unit cost ranging from 150 Euros of the former to about 600 Euros of the latter. 

The concurrent need for higher density GNSS networks and the availability of new multi-constellation multi-frequency GNSS chipsets has created the opportunity for a research project named GREAT (next generation Gnss REference stATion), funded by the European Agency for the Space Programme (EUSPA) as part of the “Fundamental Elements” call 2019 (grant. N. GSA/GRANT/04/2019/GREAT). The project has been carried out by a consortium of four SMEs based in Italy (SpaceExe Srl, project coordinator—www.spaceexe.com, accessed on 28 June 2023—and YetItMoves Srl—www.yetitmoves.it, accessed on 28 June 2023), France (Exagone Réseau Teria—www.reseau-teria.com, accessed on 28 June 2023, hereinafter referred to as Teria) and Belgium (M3Systems Belgium—https://m3systems.eu/en/m3-systems-belgium/, accessed on 28 June 2023). Its main goal is the development, test and demonstration of an innovative GNSS reference station, capable of offering performance levels similar to the ones of the current geodetic grade stations but with the following distinguishing features:

- costs (in terms of equipment, setup and maintenance) up to a quarter of the present standard.
- single integrated device including on-board cellular LTE modem and solar panel charger, in order to simplify the installation as much as possible.
- low power consumption, including all the aforementioned components, respect to the standard (6–8 Watts), in order to allow the receiver to be easily powered with solar panels and small backup batteries when installed in remote areas.

In order to simplify the receiver architecture as much as possible (i.e., to reduce its computational load and power consumption), in the GREAT system several functionalities are moved from the receiver itself to a software tool for real-time remote data acquisition and processing, which can be installed on a processing server, and that implements the following functionalities:

- Real-time data acquisition from a network of GREAT stations (or from external third-party receivers).
- Near-real-time spoofing and jamming detection.
- raw GNSS data quality measurement using third party software like GNUT-ANUBIS Free 2.3 [16].
- Computation of periodic (e.g., daily) station displacement based on a network adjustment approach.
- Generation of alerts in case of anomalies in telemetry, GNSS data quality and abnormal displacements of the antenna (e.g., caused by physical movements of the monumentation structure or of the ground where the antenna is installed).
- Generation of hourly RINEX 3 or 4 files (Hatanaka compressed and zipped) containing the observables of each receiver part of the network, and their publication through HTTPs or FTPS protocols toward the final users.
- Computation of the hash string of each hourly RINEX file (a 512 bytes string that acts as a “signature” and that univocally identifies the original file) and its storage on an external blockchain implementation. In this way, every final user at every time can compare the hash string saved on the blockchain with the one that he/she can compute from the RINEX file downloaded by the server, in order to be sure that the RINEX file was not modified after its creation.

This paper aims at summarizing the main outcomes of the project, which has reached its conclusion in May 2023. We present the architecture of the developed system, with special focus on its innovative and distinguishing features; moreover, we provide a description of the field test campaign which has been realized in the last months of the project and the main achieved results.

## 2. Related Works

Currently, geodetic grade GNSS receivers exploited in the realization of permanent stations are mainly COTS products commercialized by large global suppliers, such as Leica Geosystems, Trimble, Topcon or Septentrio, just to mention the most important ones. The reason is related to the need to have high performance and reliable instrumentation suitable to work continuously, possibly for several years, with the minimum maintenance possible. However, in the last few years new players have entered the market, both from Europe (companies such as Stonex) and the Far East (e.g., Tersus GNSS), offering advanced products at relatively lower prices with respect to the standard.

Besides such commercial products, it is worth mentioning that several research projects, mainly financed by European agencies, were dedicated in the last few years to the development of innovative GNSS receivers. Among the others, we can mention here the eMAPs project [17], financed by EUSPA and aimed at developing a low-cost, cloud-based multi-sensors platform which hybridizes data generated by a multi-frequency multi-constellation GNSS receiver, or BB-PROC [18], financed by the European Space Agency (ESA) under the NAVISP program, whose objective was the development of a prototype of a GNSS sensor tailored to automotive applications. 

When dealing specifically with GNSS reference stations, it is important to mention the RIMS V2 Breadboard (R3B) project [19], financed by ESA and carried out in 2016–2017. The objective of the project was the design and development of an innovative Reference Station Prototype integrating two state-of-the-art multi-constellation multi-frequency (MC/MF) GNSS receivers. Finally, we can mention the recent GAMMA project [20], financed by EUSPA, targeted at the development of a multi-frequency multi-purpose high-end GNSS antenna with spoofing and jamming rejection features, specifically tailored for the implementation of reference stations.

However, among all mentioned projects, GREAT presents several distinctive features, such as the integration on a single electronic board of all components for power supply and data transmission (which is probably unique in the panorama of GNSS reference stations), and the attention paid to the minimization of power consumption, which makes the system suitable for installation in remote areas. 

## 3. Materials and Methods

This section reports a description of the GREAT GNSS system, including its hardware and software components. Indeed, the goal of GREAT is not only to develop a GNSS receiver but a comprehensive system, including both hardware and software modules. The following subsections describe in detail each part.

### 3.1. GREAT GNSS Receiver

The GREAT GNSS receiver is designed to integrate all the functionalities needed to operate a GNSS reference station, without the need for external devices or components, such as battery chargers or telecommunication devices. Moreover, it includes a number of external interfaces, useful to be easily integrated in existing or future systems.

In particular, the following functions are integrated:

- power control and battery charging via photovoltaic panels.
- wireless communication (4G + ISM 868 MHz).
- monitoring grade accelerometer.
- analog input channels for external sensors (standard 0–20 mA).
- data storage (SD + Flash memory).

**Figure 1:**
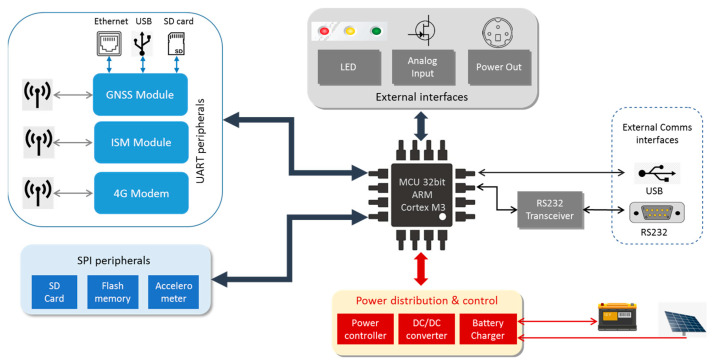
GREAT receiver hardware architecture.

**Figure 2:**
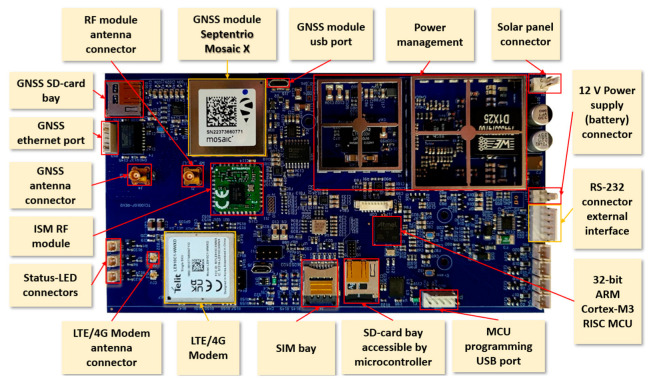
GREAT GNSS receiver board with integrated components.

The hardware architecture is shown in Figure 1. All the peripherals and I/O connectors shown in the picture are integrated into a single printed circuit board (PCB), except of course, the lead acid battery and photovoltaic panel. Therefore, the so-called “GREAT GNSS receiver”, shown in Figure 2, integrates in a single and compact device all the components needed to easily run a reference station shown in Figure 1.

**Figure 3:**
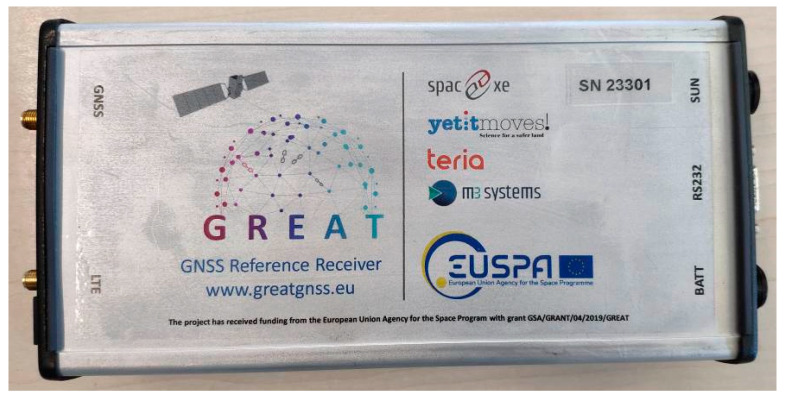
GREAT GNSS receiver in its prototypal aluminum enclosure.

Figure 3 shows the board inside its enclosure, specifically realized for the project. In order to minimize the size of the enclosure, we set up the following interfaces: status LEDs, battery input, solar panel input, Serial RS232 I/O, Ethernet I/O, RF GNSS antenna input and RF LTE antenna input. Other interfaces (PPS, Analog I/O, USB) are on the board and can be brought out of the enclosure if needed.

The Micro-Controller Unit (MCU) is a 32-bit ARM Cortex M3 based architecture. It combines low power consumption and several internal peripherals and interfaces. It is an engineering trade-off between other more powerful and energy consuming architectures (such as the ones based on computer modules and Real-Time Operating Systems—RTOS) and ultralow power MCUs.

The GNSS core is a Septentrio Mosaic X5 chipset [15], which was selected among different competitors available on the marked at the time of design (e.g., Ublox ZED-F9P [13] or ST Microelectronics Teseo V [21]) and which resulted the best tradeoff between costs and power consumption on one hand and the capability of tracking all the main frequencies (at least L1/E1/G1/B1C plus L2/E5b/G2 plus L5/E5a/B2a) of the principal GNSS constellations (GPS, GLONASS, GALILEO, BEIDOU) on the other. The main features of the chipsets considered for integration are listed in the following Table 1.

The power distribution and control unit are a key feature of the GREAT receiver. It integrates in the same PCB the power distribution and the module to charge an external 12 V lead acid battery, using a maximum-power-point-tracking (MPPT) battery charger powered by a photovoltaic (PV) panel. The lead acid battery is the main source of power of the GREAT receiver, even during nighttime; the PV panel is used to recharge (and/or maintain charged) the battery during daytime. In the most energy consuming case (all constellations on, 1 Hz update rate, LTE and wireless modem on) measured average power consumption is about 2.5 W. As demonstrated by the field tests described in the following sections of this paper, the device can work with a 12 V/36 Ah lead acid battery and a 100 Wp PV panel. The autonomy of this configuration, in case of fully charged battery and PV panel failure is (12 V × 36 Ah)/2.5 W ≅ 172 h ≅ 1 week. The daily power consumption of the receiver is about 2.5 W × 24 h = 60 Wh; it means that the PV can completely recharge the daily consumption in less than one peak sun hour (also considering 80% conversion efficiency only). In accordance with the National Renewable Energy Laboratory, average peak sun hours across the US (Source: NREL 2018 October solar data), the worst case in US during winter is in Alaska, where the peak sun hours are 1.78 per day. Therefore, we can consider that this configuration can be balanced even in the worst weather conditions. Although the PV technology is mature and the PV panels failure is unlikely, it is always possible to include (when available) a wired power source (any industrial AC/DC power converter) in parallel to the batteries, for further redundancy. It is worth noticing that the MCU periodically transmits telemetry data (i.e., solar panel voltage and battery voltage), which are useful for remote diagnostic of the device, for verifying any performance degradation and for planning any preventive maintenance. The integration of a solar charger inside a receiver is a quite novel approach in the GNSS reference station panorama. In fact, all the consortium’s known GNSS receivers suitable for implementing a continuously operating reference station (CORS) do not include a solar charger; this approach has the advantage to reduce the costs, complexity and cable routing inside the installation cabinet. 

Besides standard physical interfaces (RS232 and USB), the board hosts integrated components for wireless data transmission, among which an ISM modem working on the 868 MHz band (useful for local data transmission) plus an LTE modem, which represents the most mature technology for cellular data transmission at the time of design of the system. The front-end of the ISM modem integrates a narrow band SAW filter for certification and intra-board EMI reduction, so that the second harmonic on the 868 MHz band power amplifier does not interfere with the L1 (1575 MHz) GNSS band. For what concerns the cellular connectivity, among the different competitors, a Telit LT910-WW modem [22] was selected for integration. Such CAT1 LTE module can be easily programmed in order to transmit all data generated by the GREAT receiver, as to access the receiver from remote for its configuration. Moreover, it allows transmitting in real-time the telemetry information related to the cellular signal strength and to the bit-error-rate. Finally, the module can be reached with SMSs with an independent software interface, which allows getting diagnostic data as like to perform simple operations such as module reboot.

The board integrates a tri-axial digital MEMS accelerometer (model Analog Devices ADXL355 [23]), which can be programmatically turned on/off at MCU level and whose raw data can be logged and transmitted.

The GREAT receiver is equipped with an industrial grade 16 GB micro-SD card, used to log a copy of all acquired data. The system can track several telemetry parameters related to the storage, such as the average write time or the number of cyclic redundancy check (CRC) errors that occurred during writing operations.

Finally, the board integrates eight analog inputs used to easily integrate any third party 4–20 mA standard sensor (for example temperature, humidity, pressure, vibrations, acceleration, strain gauge, etc.) and periodically transmit these data in the same telemetry packet. The GREAT SW platform can show and use these data for post-analysis. 

The PCB dimensions are 19 × 9.5 × 1.5 cm and external GREAT receiver dimensions are 22 × 10.5 × 4.5 cm (excluding mounting brackets/flanges and connectors). The total weight of the GREAT receiver is about 0.6 kg.

### 3.2. Acquisition and Processing Software

**Figure 4:**
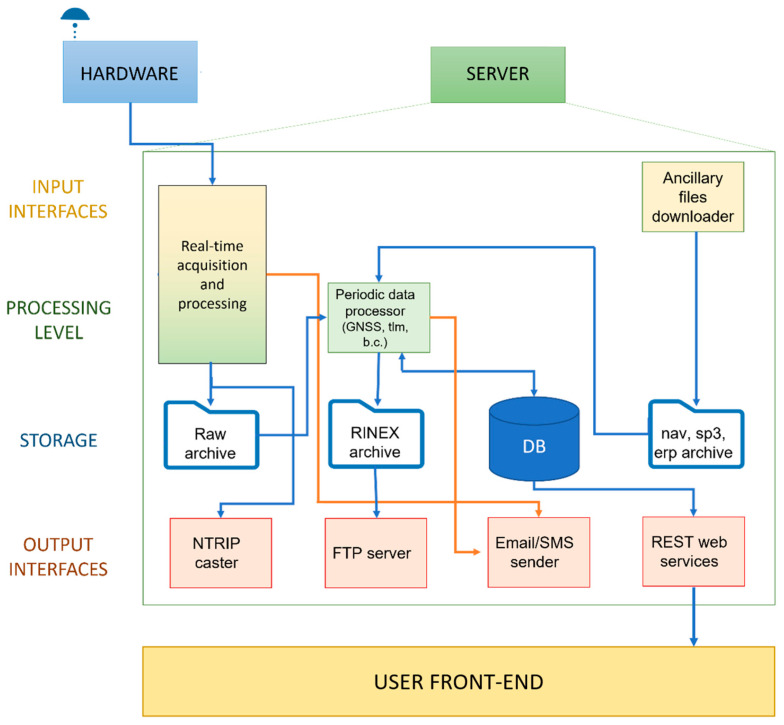
Functional architecture of the GREAT software modules.

According to the general architecture of GREAT, several functionalities of the system are not implemented at hardware level by they are demanded to software components designed to run on a remote “acquisition and processing server”. Such server module is mainly dedicated to real-time data acquisition and validation (in terms of possible spoofing and tampering, as of GNSS quality check), to their archiving and publication both as real-time (RTCM 3.2 corrections) or post-processing (RINEX 3 or 4) products. The server software also includes modules for user alerting through email and SMS. Figure 4 represents the functional architecture of the developed software.

From a functional point of view, the main modules of the software tool are the “real-time acquisition and processing” (RTAP) and the “periodic data processor” (PDP) components. 

**Figure 5:**
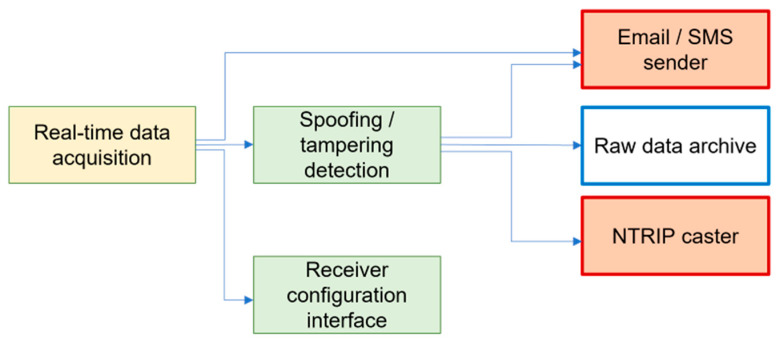
Functional architecture of the Real Time Acquisition and Processing (RTAP) module.

At each software startup, an instance of RTAP is executed for each GREAT station part of the controlled permanent network, and it implements the following actions, summarized in graphical style in Figure 5.

- It tries to establish a connection with the GREAT receiver:
  ○ If the connection is established, then it starts getting all acquired data in real-time (raw GNSS observables plus telemetry), and to interpret the GREAT binary protocol, in order to separate the different data types.
  ○ If a connection is not successful or if during the acquisition the connection is lost with the receiver, a warning message is promptly generated and sent to the “email/SMS sender” in order to warn the user in almost-real-time.
- GNSS data acquired by the previous module are passed to the “real-time spoofing detector”, which verifies the authenticity of the acquired data:
  ○ If data pass the check, then they are published in real-time through the integrated NTRIP caster and are saved into the temporary “raw data archive”.
  ○ In case of spoofing or tampering detection, then data are discarded, and an alert is raised and transmitted in almost-real-time to the “email/SMS sender”.

The spoofing detection procedure is based on the computation of several quality indexes on raw GNSS data (including variance and minimum and maximum values of C/N0 of the code pseudoranges and of Doppler measures on each tracked frequency of each satellite system) as on the detection of anomalous displacements. The routine for raw GNSS data control implemented here has been adapted from the algorithms presented in [24]. The control on the displacements is made under the hypothesis that a permanent station is supposed to be steady during time; any displacement outside a tolerance range (e.g., average position ± n·standard deviations) can be considered anomalous. Finally, the routine exploits the output of the spoofing-detection algorithm implemented at hardware level by the Mosaic X5 chipset. The implementation of the server-side anti-spoofing module is based on a customized version of the rtkrcv.exe software part of RTKLIB 2.4.3 b34 tool [25,26]. A detailed description of the algorithm, which is currently under test, is beyond the purposes of this paper, and it will be possibly discussed in a dedicated work.

**Figure 6:**
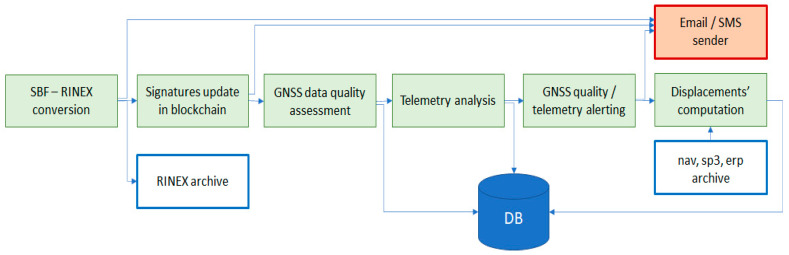
Functional architecture of the Periodic Data Processor (PDP) module.

A single instance of the PDP module manages the whole network of GREAT receivers. Every hour, it executes in sequence the tasks shown in Figure 6:

- It converts the binary files containing the raw GNSS observables of all stations part of the network into hourly RINEX files with observables sampled at 1 Hz (format 3.04 or 4.0, Hatanaka compressed and g-zipped) and archives them in the filesystem. Such an archive can then be published to possible final users with an FTP implementation. The module warns the user with email/SMS in case a specific conversion process fails or in case of unavailability of an expected binary file.
- It computes the SHA-512 hash string [27] of each generated RINEX file and saves them into a blockchain implementation. In detail, we have used an Amazon AWS Quantum Ledger Database (QLDB) instance [28] for demonstration purposes, but alternative solutions can be easily exploited. The SHA-512 hash string univocally identifies the content of the RINEX file since every possible modification (even of a single byte) of the content drives to the computation of a completely different hash. Therefore, every user can verify the absence of any data tampering on the file after its creation simply computing the SHA-512 hash of the file he/she owns and comparing it with the one computed by the processing server software just after the file creation and retrieved from the blockchain. Moreover, storing the hash string on a blockchain implementation guarantees that no-one, even the owner of the processing software, can modify the initial value of the hash string after its initial upload.
- It computes a set of quality parameters related to the raw GNSS data acquired in the last hour, such as percentage of acquired over expected GNSS epochs and observables, percentage of observables with cycle slips, average multipath and carrier to noise ratio. All parameters are computed for each frequency of each tracked satellite system. In order to generate trustable results, quality indexes are computed exploiting an instance of the G-NUT Anubis Free software [16], which is embedded in the PDP.
- It computes the average telemetry parameters (battery voltage, solar panel voltage, number of CRC errors and average SD-card writing time, cellular signal strength, etc.) belonging to data acquired in the last hour.
- It generates possible warning messages in case of detection of anomalous values on GNSS quality and telemetry. The software compares the last updates of all computed variables with a set of thresholds defined by the user. If any threshold is exceeded, then a message is generated and transmitted to the user by email.
- It computes the accurate and precise antenna phase center displacement in the last period. Such a computation is based on a static-differential approach working with double differenced carrier phases observables [29,30]. The computation is realized exploiting an upgraded version of the DISPLAYCE software tool developed by YetItMoves [31]. The development activity carried out in the framework of the GREAT project and in the previous H2020 TURNkey project [32] allowed to extend the functionalities of the original DISPLAYCE software described in [31] and initially meant to work with single frequency data, introducing the software routines required to handle multi-frequency GNSS observables, thus allowing to manage regional scale baselines up to 200–300 Km. The most important features added to the computation software include:
  ○ the exploitation of the ionospheric-free linear combinations of GNSS observables, which are computed on the input L1, L2 and L5 observables and used in the positioning process.
  ○ the exploitation of solid tide and ocean tide loading (OTL) models, the latter exploiting the OTL parameters computed for each specific location by the Onsala Ocean Tide Loading provider [33].
  ○ the possibility to perform a simple “network adjustment” strategy on the results of baseline processing, that is to compute the differential displacements of each GREAT station with respect to two or more reference stations, whose a priori coordinates are known with an high degree of confidence (e.g., stations belonging to national or international permanent station networks such as IGS or EUREF), and then to estimate the absolute coordinates of the station with a least square approach [34].

All periodic tasks are repeated indefinitely until closing the software or interrupting the acquisition.

### 3.3. User Front-End

The GREAT client application is the third component of the system described in this paper and it represents the front end of the software tool that manages the network of permanent stations. It allows the user both to monitor the status of the stations and to modify their configuration. More in detail, it allows showing all computed variables (regarding telemetry, GNSS data quality and displacements) in graphs and table format for each station part of a monitored network. Moreover, it allows the user to set all the thresholds exploited by the server-side software to raise the alerts described in the previous section.

It is designed as a web application composed of a set of graphical interfaces, accessible only by authenticated users. Since it does not implement relevant scientific or technical innovative elements, it is not described in detail in this work. We just mention that all figures of maps, graphs, etc. reported in the chapter about field validation are screenshots of the GREAT client application.

## 4. Field Validation

According to the project schedule, fourteen GREAT GNSS receiver prototypes were assembled and used in a series of laboratory and field tests. Lab tests are aimed at assessing the hardware performance in a controlled environment, in terms of tracking capabilities, GNSS data quality and spoofing/jamming detection. On the other hand, field tests aimed at demonstrating the actual feasibility of using the GREAT GNSS receivers for the implementation of a network of permanent stations. For this reason, two networks of GREAT receivers have been installed in the Île-de-France (France) and Wallonia (Belgium) regions in April 2023. In this chapter we describe the setups realized in the two testbeds. Chapter 5 summarizes the most important achieved results.

### 4.1. Île-de-France Testbed

A network of four GREAT receivers has been installed in the Île-de-France region in France, in the locations listed in Table 2:

The installation setup is identical for each site. A dedicated task of the project was aimed at selecting the most suitable antenna for the GNSS reference station implementation. A Tallysman VSP6337L [35] has been chosen, being the best trade-off between cost and quality. In fact, despite its relatively low-cost, it has a very tight (mm-level) phase centre variations (PCVs) and its calibration values for all tracked satellite systems and frequencies are available in the official ANTEX file published by the IGS [36]. This is a quite uncommon but distinguishing feature, since most of the geodetic grade antennas currently exploited for permanent GNSS stations have PCO/PCV calibration available only for the GPS L1/L2 and GLONASS G1/G2 frequencies. The antenna has been connected to the receiver thanks to a 3 m long low-loss (LC200) coaxial cable.

The antenna is monumented to the ground using a “roof pallet” structure, a solution already exploited by Teria (part of the GREAT consortium and responsible for the field tests) in its operational installations of permanent GNSS stations (see, for example, https://rgp.ign.fr/STATIONS/#chdy, accessed on 28 June 2023). The consortium is aware that such a monumentation solution does not meet the strict IGS Monumentation Design and Implementation Recommendations (https://igs.org/station-resources/#monumentation-recommendations, accessed on 28 June 2023); anyway, we considered relevant for the purpose of the project (i.e., to design and develop cost effective solutions for the implementation of reference GNSS stations) to investigate the possibility to exploit this kind of monumentation type and to quantify the achievable performance in terms of antenna stability and quality of the generated GNSS data. This solution has the advantage of being extremely low-budget, quick and easy to install. In detail, the structure used for the monumentation consisted in:

- a roof pallet with a steel structure, 1 m mast with 60 mm diameter, two force arms to support the mast on the steel structure and serrated jaws. The structure has been custom built for Teria by a local manufacturer.
- six concrete slabs.
- two triangular plates with three screws to ensure the levelling of the antenna.
- two horizontal steel racks to support the cabinet on the mast.

**Figure 7:**
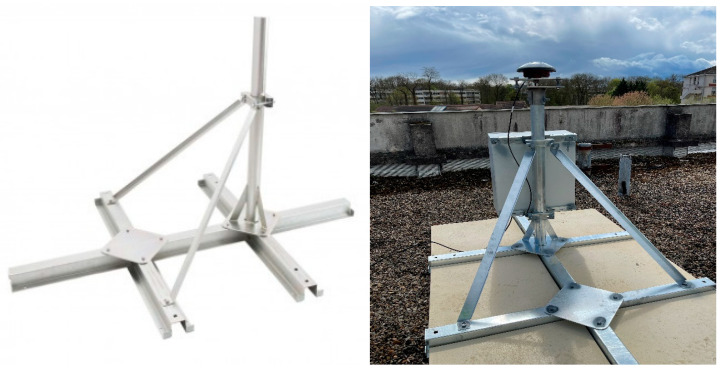
Roof pallet monumentation structure (**left** panel) and monumentation work installed in one of the sites in the Ile-de-France testbed (**right** panel).

The following Figure 7 shows the monumentation structure. The GNSS receivers are placed inside an IP66 protective cabinet, which also hosts a 12 V 34 Ah backup battery. The cabinet is connected to the basis of the monumentation structure. The receiver is powered by a 100 W photovoltaic panel, fixed to a dedicated aluminium structure placed near the antenna monumentation structure.

The assembly time of the structure for two persons is about two hours, including the installation of the cabinet, the antenna as well as the aluminum structure that supports the solar panel. It must be noted as such the installation time is significantly less than the typical time needed to realize the monumentation of permanent GNSS stations following the IGS standards.

**Figure 8:**
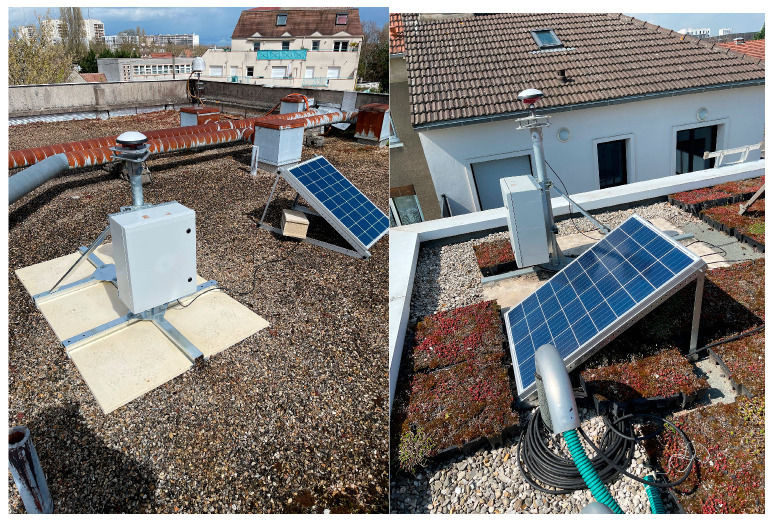
GNSS stations installed in MEL200FRA (**left** panel) and MLV200FRA (**right** panel) sites.

**Figure 9:**
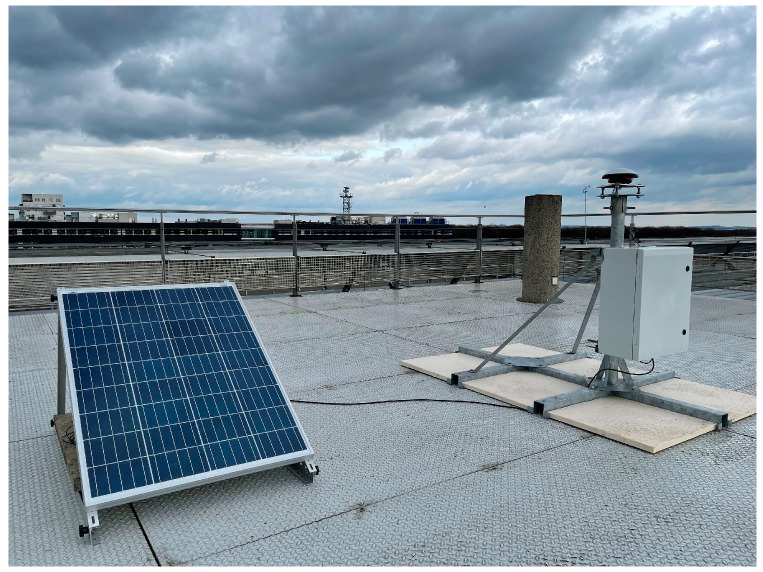
MLV200FRA station.

Figure 8 shows photographs of the installation of MEL200FRA and VIT200FRA, whereas Figure 9 shows the installation site of MLV200FRA.

Besides the GREAT stations, the network includes five “higher order” GNSS reference stations belonging to the EUREF network, whose precise coordinates are well known, which are used to compute displacements of the GREAT stations on a daily basis according to the strategy described in the previous section.

The most important information about the five “external” reference stations is summarized in Table 3.

**Figure 10:**
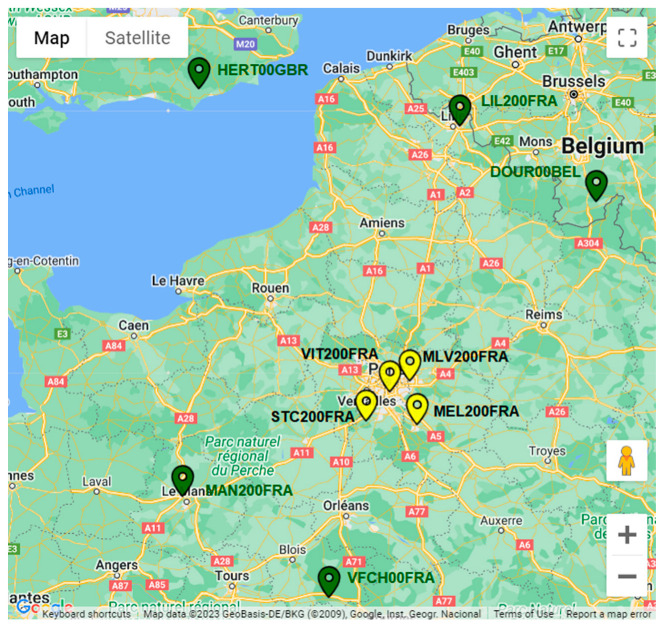
Map of the GREAT GNSS network and external reference stations in France.

Finally, Table 4 shows the inter-distance between the GREAT stations and the five external references, expressed in kilometers. The following Figure 10 shows a map of the network: stations identified by a yellow icon are the GREAT stations, whereas stations identified by a green icon are the external references. 

### 4.2. Wallonia Testbed

A second network composed by two GREAT receivers has been installed in the Wallonia region in Belgium, in the locations listed in Table 5.

**Figure 11:**
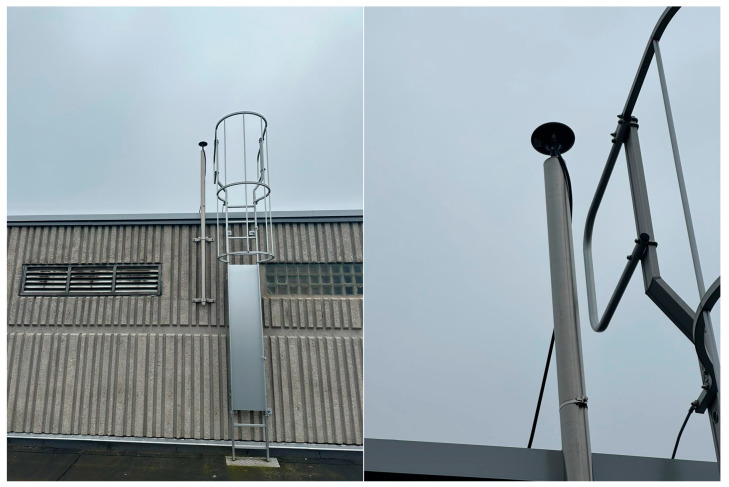
GNSS antenna installed in the LIEG00BEL site: whole structure (on the **left**) and a detail of the antenna (on the **right**).

In this testbed, the two stations are equipped with a very low-cost multi-frequency GNSS antenna, model Ardusimple ANT-3BCAL [37], which is characterized, as the Tallysman model exploited in the former testbed, by very tight PCVs (smaller than 3 mm at all elevations) and whose calibration is available in the calibration file published by the US National Geodetic Survey (NGS) [38]. In the two sites, the antennas are directly connected to the walls of the existing buildings with steel brackets, as shown in Figure 11. The same considerations made for the “roof pallet” monumentation and reported in the previous paragraph apply here. The purpose of testing this kind of really “low budget” monumentation it to investigate the achievable performance in terms of antenna stability, and therefore the possibility to exploit it in the future in the implementation of local or regional permanent stations.

The connection between the antenna and receiver is realized by exploiting a 10 m low loss (LLC200) coaxial cable.

The GREAT receivers are hosted inside the buildings and they are powered directly by the 220 V AC grid with a standard grade AC/DC converter. Such setup allows to measure possible issues on the acquired GNSS data that can be caused by ripples and disturbances of the power supply.

**Figure 12:**
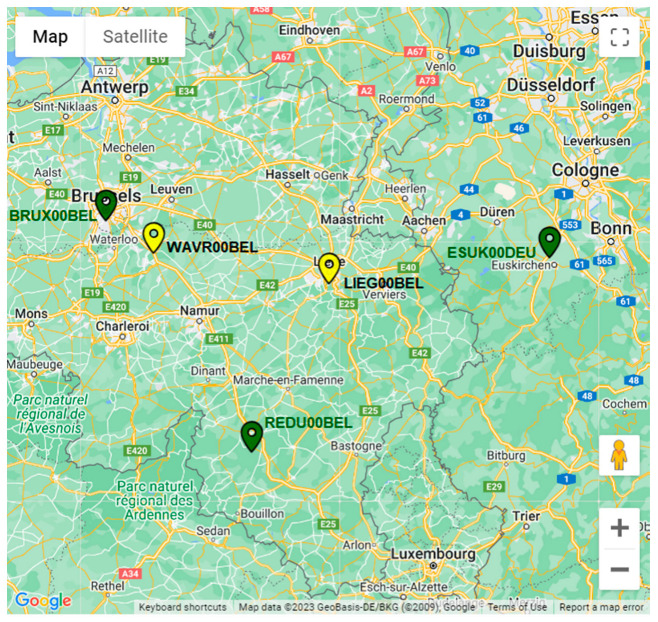
Map of the GNSS network and external reference stations in Belgium.

Similarly to the testbed in France, the network is completed by three external higher-order reference stations, part of the EUREF network and used for the computation of the precise coordinates of the GREAT stations. Details about such external stations are available in Table 6; the subsequent Table 7 shows the inter-distance between the GREAT stations and the three external references, expressed in kilometers; finally, Figure 12 shows a map of the network including the external references.

### 4.3. Field Assessment Procedure

The aim of the field validation is to assess the performance of the entire system (hardware, remote data transmission and software) in terms of continuous station operation and of achieved GNSS data quality. It must be noted that, at the time of writing this document, only preliminary results can be reported, given that the hardware has been installed only for a few months and a complete assessment of the system should require at least one year of continuous operation, in order to detect all possible long-term issues or misbehaviors.

Nevertheless, in this paper we would like to present an assessment methodology which will be extended to a longer period. In detail, the following key performance indicators (KPIs) are considered to quantify the performance of the system.

KPIs related to the station operation continuity:

- Number of gaps in real-time data transmission with duration longer than 120 s.
- Average and maximum length of data gaps.
- Minimum battery voltage.
- Average and minimum cellular signal strength (as recorded by the embedded LTE modem).

KPIs related to the GNSS data quality:

- Average and minimum percentage of acquired/expected epochs computed on hourly basis.
- Average and minimum percentage of acquired/expected observables computed on hourly basis and for each frequency of each satellite system.
- Average and maximum percentage of cycle slips computed on hourly basis, for each frequency of each satellite system.
- Average and maximum multipath on code-pseudo-ranges computed on hourly basis, for each frequency and satellite system.
- Average and minimum C/N0 computed on hourly basis, for each frequency of each satellite system.
- Root mean square (RMS) of the daily 3D displacements of the station computed with the network adjustment process described in Section 3.2.

Moreover, we have tested the hardware prototype power consumption in the following operating conditions:

- GNSS data acquisition at 1 Hz (GPS + GLO + GAL, all frequencies enabled) and their remote real-time transmission to the processing server using the internal LTE modem (called “GREAT mode” in the following).
- GNSS data acquisition disabled with LTE module powered on (idle mode).
- GREAT mode with disabled remote transmission (LTE module powered off).
- Idle mode with the LTE module powered off.

and using the following KPIs:

- The maximum current found multiplied by the maximum voltage.
- The maximum current found multiplied by the minimum voltage.
- The minimum current found multiplied by the maximum voltage.
- The minimum current found multiplied by the minimum voltage.

A specific device has been set up to automatically detect the maximum and minimum current consumed within ten minutes, whilst, for voltage, an AVOmeter has been used and the maximum and the minimum voltage has been found by inspection methodology. 

## 5. Results

This section summarizes the results achieved in the experimentation at the two testbeds in France and Belgium, in terms of the KPIs listed in Section 3.3. Results refer to a dataset acquired during the period from 15 April to 4 May 2023 (20 consecutive days) by all installed stations.

### 5.1. Île-de-France Testbed

Table 8 summarizes the results achieved in terms of station operation continuity.

**Figure 13:**
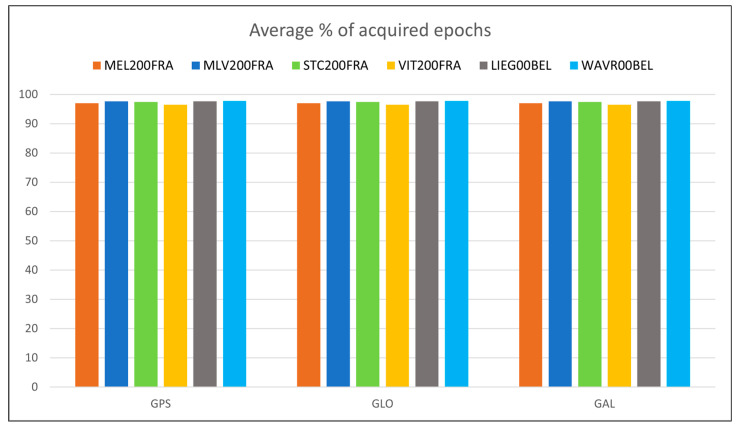
Percentage of acquired GNSS data epochs of each satellite system for each station in the Ile-de-France and Wallonia testbeds.

**Figure 14:**
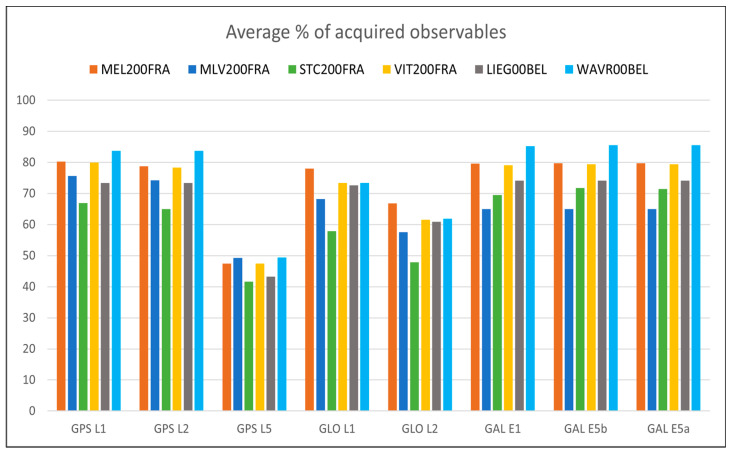
Percentage of acquired GNSS data observables of each tracked satellite system/frequency for each station in the Ile-de-France and Wallonia testbeds.

**Figure 15:**
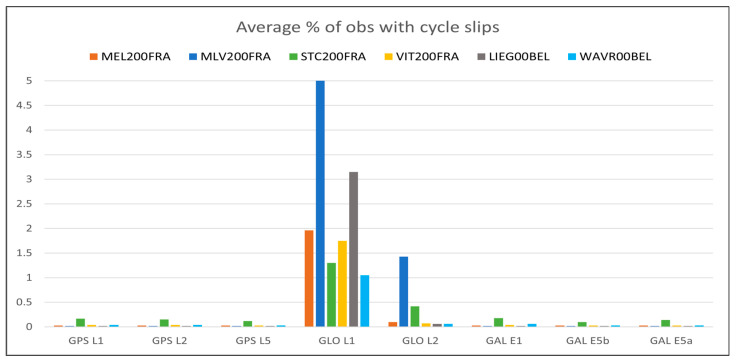
Percentage of acquired GNSS data observables with cycle slip of each tracked satellite system/frequency for each station in the Ile-de-France and Wallonia testbeds.

**Figure 16:**
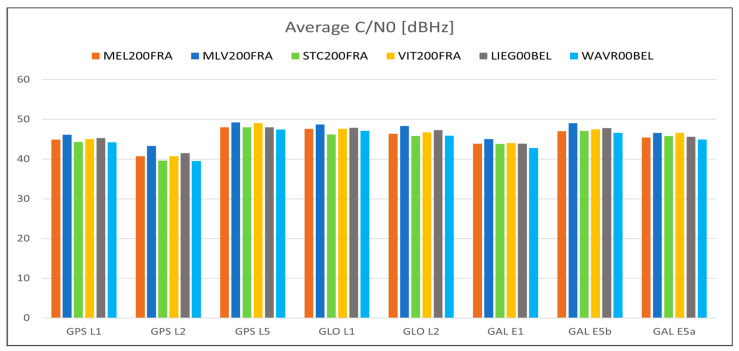
Average carrier-to-noise-ratio of each tracked satellite system/frequency for each station in the Ile-de-France and Wallonia testbeds.

**Figure 17:**
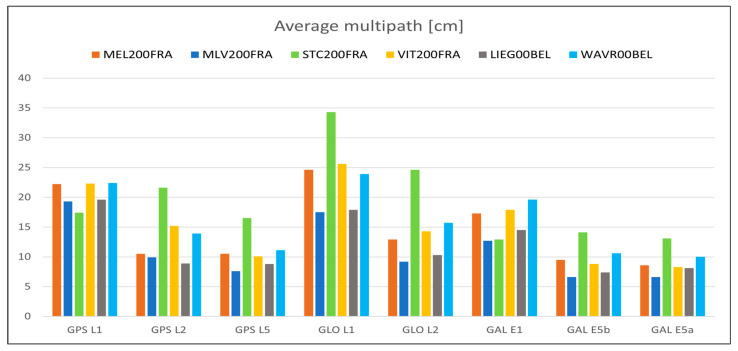
Average multipath of each tracked satellite system/frequency for each station in the Ile-de-France and Wallonia testbeds.

For what concerns the GNSS data quality, the following Figure 13, Figure 14, Figure 15, Figure 16 and Figure 17 summarize in graphic form the achieved average results of the four GREAT stations (first four vertical bars in each group); the same figures also report the analogous parameters measured in the Wallonia testbed, as described in Section 5.2. In detail, Figure 13 shows the average percentage of acquired vs. expected epochs for each satellite system, Figure 14 shows the average percentage of acquired observables for each tracked signal of each satellite system, Figure 15 shows the percentage of observables with cycle slips, Figure 16 shows the average carrier-to-noise ratio and, finally, Figure 17 shows the average multipath. All values plotted here are reported in tabular form, together with the minimum values obtained in each test, in Appendix A, Table A1 (results on GPS data), Table A2 (Glonass) and Table A3 (GALILEO).

Table 9 reports the achieved repeatability (RMS) of the displacements of the stations.

**Figure 18:**
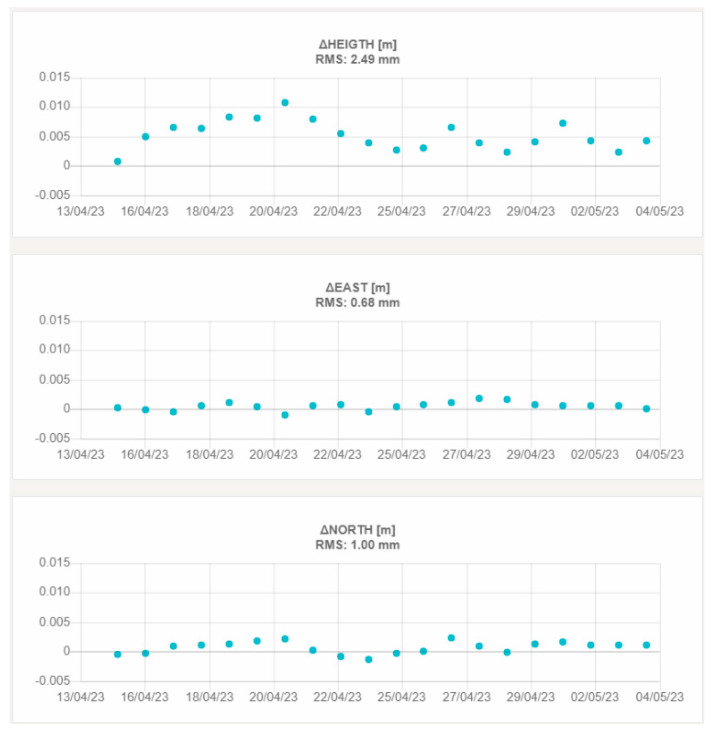
3D displacements of VIT200FRA. The upper graph shows the vertical displacement, the central graph the east–west displacement and the lower graph the north–south displacement.

**Figure 19:**
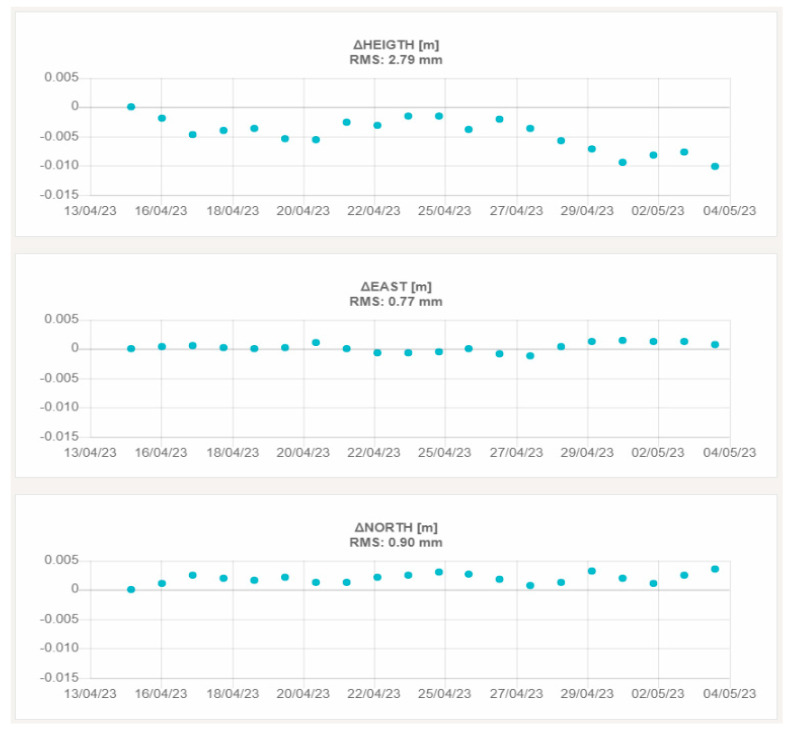
3D displacements of STC200FRA. The upper graph shows the vertical displacement, the central graph the east–west displacement and the lower graph the north–south displacement.

Figure 18 and Figure 19 report, as an example, the graphs showing the height and planimetric components of the displacements of VIT200FRA and STC200FRA, which are representative of the behavior of the remaining stations.

### 5.2. Wallonia Testbed

The following Table 10 summarizes the results achieved in terms of station operation continuity.

The previous Figure 13, Figure 14, Figure 15, Figure 16 and Figure 17 summarize in graphic form the achieved average results for the two GREAT stations in terms of GNSS data quality assessment (last two vertical bars in each group in each graph).

Table 11 reports the achieved repeatability (RMS) of the displacements of the stations.

**Figure 20:**
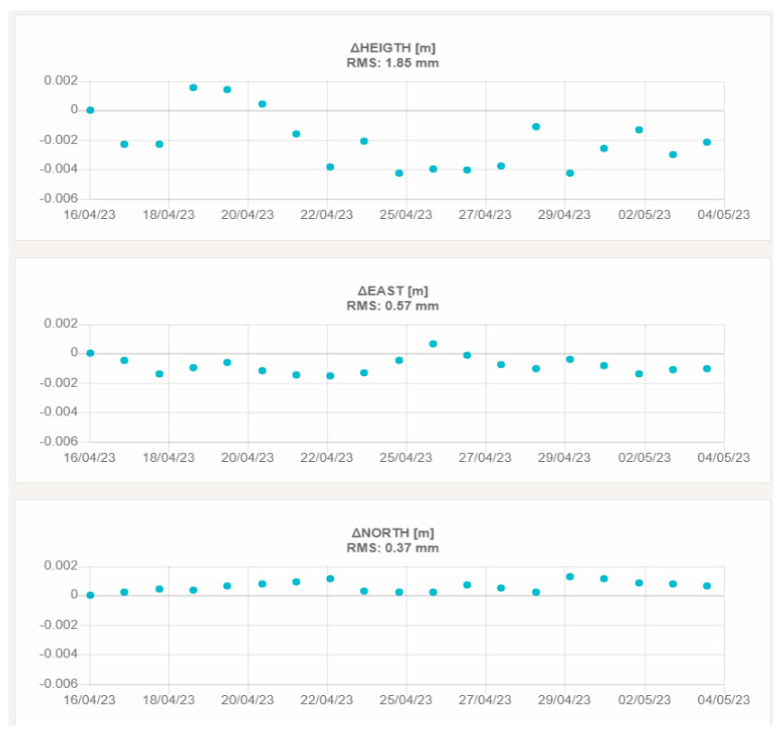
3D displacements of LIEG00BEL. The upper graph shows the vertical displacement, the central graph the east–west displacement and the lower graph the north–south displacement.

**Figure 21:**
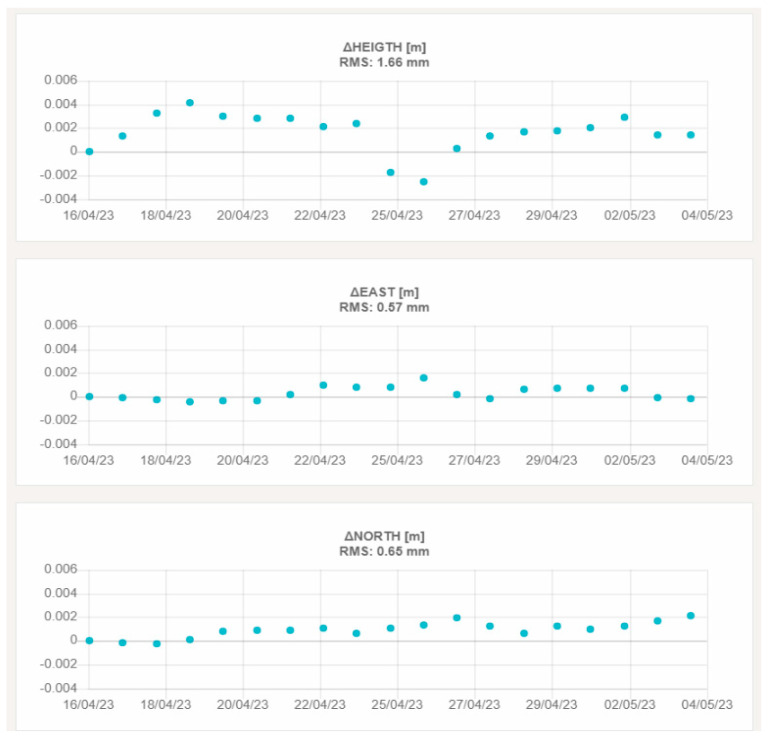
3D displacements of WAVR00BEL. The upper graph shows the vertical displacement, the central graph the east–west displacement and the lower graph the north–south displacement.

Figure 20 and Figure 21 show the graphs with the height and planimetric components of the displacements of LIEG00BEL and WAVR00BEL, respectively.

For what concerns the test of the hardware power consumption, as expected the functioning modes a–d described at the end of Section 4.3 produced different processing behaviors, hence different power consumptions, summarized in Table 12.

### 5.3. Analysis of the Results

By analyzing the results achieved in the two testbeds in terms of telemetry (items reported in Table 8 and Table 10), it is possible to highlight the following points:

- Telemetry data acquired in the first month after installation demonstrate that all stations were able to work with a high degree of continuity: on average, the stations transmitted data for more than 96.5% of the time (worse case of VIT200FRA) and there was only a reduced number of interruptions: apart from VIT200FRA, the other five stations had an average interruption less than 2 min and the number of interruptions per day longer than 2 min is limited to less than one, on average. In all cases, the maximum interruption time is less than 30 min. This evidence proves that there is no interference of GNSS toward the LTE module, which is mounted on the same electronic board. Moreover, the choice of using an omni-directional LTE antenna placed inside the protective cabinets guaranteed enough signal strength to avoid relevant interruptions in data transmission.
- For what concerns the power supply subsystem based on photovoltaic panels and backup batteries (in the first testbed), the minimum measured value of the battery voltage (12.23 Volts measured by STC200FRA) proves that it is correctly dimensioned given the device power consumption and the installation site.

For what concerns the performance of the system in terms of GNSS data quality, we can underline the following:

- The results summarized in Figure 13, Figure 14, Figure 15, Figure 16 and Figure 17 and in the tables in Appendix A show that in all cases the receivers provided a high percentage of acquired over expected observables on all GPS and GALILEO frequencies. The average value is higher than 67% in case of GPS L1, 65% in case of GPS L2, 69% and 71% in case of GALILEO E1 and E5a/b, respectively. It must be remarked that the percentage of observables in the GPS L5 frequency is less than the one in L1 and L2 because only modernized GPS satellites (Block IIF or newer) transmit such a signal: in May 2023, only 18 operational satellites over 31 are delivering the L5 signals.
- The percentage of cycle slips is negligible, i.e., average values are always significantly lower than 1% on all stations and tracked signals. A high amount of cycle slips indicates a possible issue with the power supply (high ripples or an oscillating voltage are not correctly managed by the GNSS chipset) or electromagnetic or physical disturbances in the environment near the installation site (both factors affecting the continuous tracking of the satellite). This is not the case in both test sites, featuring different power supply subsystems (photovoltaic panels in France and AC/DC converters in Belgium). Moreover, this result demonstrates that the LTE modem (which is always turned on for real-time data transmission) does not interfere with the GNSS receiver.
- The average C/N0 is always higher than 39.5 dBHz and commonly higher than 43 dBHz: this is proof of the correct functioning of the GNSS antenna and of the adequacy of the selected coaxial cables.
- Finally, the detected multipath is acceptable on all sites (average values always less than 23 cm), which is proof of the suitability of the environment near the selected installation sites. Moreover, the low multipath detected over long periods of time and on all tracked frequencies is proof of the adequate performance of the selected antenna models, despite their low-cost. In general, the comparison between the results achieved with the two antenna models exploited in the two testbeds, with identical receivers points out that, even with a very low-cost GNSS antenna like the ArduSimple ANT3B-CAL, the quality of the results is good and comparable with the one of a medium range antenna like the Tallysman VSP-6337L.
- Data reported in Table A2 and Table A5 and the graph about the cycle slip percentage (Figure 15) demonstrate that the performance of GLONASS tracking is sensibly degraded, in particular in terms of higher number of cycle slips on L1 frequency: this applies to all installation sites (even if it is particularly evident in MLV200FRA), despite of the exploited antennas and power supply type. The reason for this finding is currently under investigation.

The third aspect that was investigated in this field validation is the repeatability of the displacement measures (summarized in Table 9 and Table 11 for the two testbeds). Looking at the achieved results, it is interesting to note that the RMS on 20 subsequent daily solutions is always less than 1.0 mm in planimetry and 3.0 mm in the height component. This result demonstrates the performance of the custom developed GNSS processing software when working with regional baselines up to 300 Km. Moreover, the preliminary results achieved in the French testbed suggest that the “roof pallet” monumentation solution proposed by Teria is suitable for a certain range of applications. If confirmed by long-term tests, this finding opens to the possibility to exploit such a relatively simple installation system in a wide variety of contexts, where the realization of more invasive monumentation types is not feasible or not practical.

For the power consumption test, as expected, the highest values were measured when the device was in GREAT mode with transmission, since most of the device peripherals were used. However, it is interesting to note that such a maximum value is about 3 W, which is really low if compared with standard COTS geodetic receivers.

## 6. Discussion

The main objective of the GREAT GNSS project was the development of a brand new GNSS receiver suitable for implementing a reference station, able to achieve the same performance of the current state-of-the-art geodetic-grade receivers but with distinguishing features in terms of low-cost and low power requirements. For this reason, the project consortium has developed an integrated system composed of a hardware module (the GNSS receiver itself) plus a software component, which implements real-time data acquisition, quality and integrity checks, storage and real-time transmission of the received raw GNSS data to external software used to provide RTCM corrections to the users. 

The prototypes developed during the project were used to realize two networks installed in France and Belgium, with the objective of quantifying the performance of the entire system, composed by GNSS receiver, cost-effective multi-frequency antenna and low budget monumentation solutions. One of the tasks of the project was to understand if alternative (not matching the international standards) monumentation solutions are suitable for a certain range of applications and in particular for the densification of existing geodetic networks. The performance of the custom developed acquisition and processing software tool was assessed too.

Even if the consortium is aware that an exhaustive assessment of the system will require at least one year of continuous functioning in operational conditions (and thus it will last well beyond the end of the project, which finished in May 2023), the results achieved in the field validation (from April to May 2023), as summarized in the previous section, can allow to draw the first relevant conclusions.

The field validation will continue in the coming months in order to investigate elements such as the power supply adequacy during the winter period at relatively high latitudes and the long-term repeatability of the measures (a point affected by both the hardware installation and the correct implementation of atmospheric delays estimation algorithms in the processing software) as the reliability of the data transmission means.

In particular, it is important to note that the GREAT stations installed in the French testbed are co-located with existing reference stations part of a commercial network. Such stations are realized with geodetic-grade instrumentation, monumentation compliant with the IGS standards and their data are processed with state-of-the-art software tools, such as Bernese. In this scenario, we foresee to realize a “long-term” comparison between the results achieved with the GREAT system and a benchmark, aimed at assessing the achieved performance in the same environmental conditions.

## Figures and Tables

**Table 1:** Characteristics of the GNSS chipsets considered for integration in the GREAT receiver.

GNSS Features	U-BLOX F9P	Septentrio MOSAIC X5	ST TESEO V
Tracked constellations	GPS—GALILEO—GLONASS—BEIDOU (plus other)	GPS—GALILEO—GLONASS—BEIDOU (plus other)	GPS—GALILEO—GLONASS—BEIDOU (plus other) ^1^
Tracked GPS signals	L1, L2C	L1, L2P, L2C, L5	L1, L2C, L5
Tracked GALILEO signals	E1, E5b	E1, E5a, E5b, E5 AltBoc, E6	E1, E5a, E5b, E6
Tracked GLONASS signals	G1, G2	G1, G2, G2P, G3 CDMA (future use)	G1, G2
Tracked BEIDOU signals	B1I, B2I	B1I, B1C, B2a, B2I, B3	B1I, B2I, B2A
Max sampling rate	20 Hz	100 Hz	Not specified
Physical and electronics			
Power consumption	0.3 W typical, 0.4 W peak	0.6 W typical, 1.1 W max	0.8–1.0 W
Availability as chipset	Yes	Yes	Available on modules of third-party producers ^2^
Physical dimension	17.0 × 22.0 × 2.4 mm	31 × 31 × 4 mm	22.0 × 17.0 × 3.15 mm
Environmental			
Operating temperature	−40–+85 °C	−40–+85 °C	−40–+85 °C
Storage temperature	−40–+85 °C	−55–+85 °C	−40–+90 °C
Cost (May 2021)			
Unit cost (May 2021)	175.9 EUR (1 pcs) to 155.2 EUR (>50 pcs)	About 600 EUR	Not officially available

^1^ According to paragraph 3.1 of https://www.st.com/resource/en/datasheet/sta8100ga.pdf (accessed on 28 June 2023), not all satellite systems/frequencies can be tracked at the same time: it is possible, for example, to install firmware versions that allow to track all frequencies of GPS + GALILEO (but no signals of GLONASS and BEIDOU), or two frequencies for three satellite systems (GPS + GLONASS + GALILEO or GPS + GLONASS + BEIDOU). ^2^ https://www.quectel.com/product-tag/teseo-v-chip/ (accessed on 28 June 2023).

**Table 2:** GREAT stations installed in the Ile-de-France region.

Station Name	Location	Latitude [°]	Longitude [°]	Height [m]	Details
MEL200FRA	Melun	48.539450 N	2.672366 E	129.1	Installation on the roof of a concrete building (site of an old hospital)
MLV200FRA	Champs-sur-Marne	48.841070 N	2.6587478 E	159.6	Installation on the roof of a steel and glass structure (site of the ENSG school)
STC200FRA	Saint-Chéron	48.556946 N	2.117378 E	182.4	Installation on the roof of a concrete building (site of a local technical service society)
VIT200FRA	Vitry-sur-Seine	48.773920 N	2.375548 E	144.2	Installation on the roof of a concrete building (site of Teria headquarter)

**Table 3:** EUREF stations used for network adjustment of the GREAT stations installed in the Ile-de-France region.

Station Name	Location	Details
DOUR00BEL	Dourbes (BEL)	https://www.epncb.oma.be/_networkdata/siteinfo4onestation.php?station=DOUR00BEL (accessed on 28 June 2023)
HERT00GBR	Hailsham (UK)	https://www.epncb.oma.be/_networkdata/siteinfo4onestation.php?station=HERT00GBR (accessed on 28 June 2023)
LIL200FRA	Lille (FRA)	https://www.epncb.oma.be/_networkdata/siteinfo4onestation.php?station= LIL200FRA (accessed on 28 June 2023)
MAN200FRA	Le Mans (FRA)	https://www.epncb.oma.be/_networkdata/siteinfo4onestation.php?station= MAN200FRA (accessed on 28 June 2023)
VFCH00FRA	Villefranche-sur-Cher (FRA)	https://www.epncb.oma.be/_networkdata/siteinfo4onestation.php?station= VFCH00FRA (accessed on 28 June 2023)

**Table 4:** Inter-distance between each GREAT station and the five external references in the Ile-de-France testbed [Km].

	DOUR00BEL	HERT00GBR	LIL200FRA	MAN200FRA	VFCH00FRA
MEL200FRA	222.386	308.953	233.093	195.579	155.692
MLV200FRA	201.525	277.539	201.059	201.875	183.751
STC200FRA	248.348	287.325	240.285	157.423	143.514
VIT200FRA	217.921	275.274	211.824	184.611	171.644

**Table 5:** GREAT stations installed in the Wallonia region.

Station Name	Location	Latitude [°]	Longitude [°]	Height [m]	Details
LIEG00BEL	Liege	50.582692 N	5.566598 E	301.7	Installation on the roof of a concrete building
WAVR00BEL	Wavre	50.689269 N	4.615405 E	184.6	Installation on the roof of the M3S Belgium headquarter

**Table 6:** EUREF stations used for network adjustment of the GREAT stations installed in the Wallonia region.

Station Name	Location	Details
BRUX00BEL	Bruxelles (BEL)	https://www.epncb.oma.be/_networkdata/siteinfo4onestation.php?station=BRUX00BEL (accessed on 28 June 2023)
EUSK00DEU	Euskirchen (GER)	https://www.epncb.oma.be/_networkdata/siteinfo4onestation.php?station=EUSK00DEU (accessed on 28 June 2023)
REDU00BEL	Redu (BEL)	https://www.epncb.oma.be/_networkdata/siteinfo4onestation.php?station= REDU00BEL (accessed on 28 June 2023)

**Table 7:** Inter-distance between each GREAT station and the five external references in the Wallonia testbed [Km].

	BRUX00BEL	EUSK00DEU	REDU00BEL
LIEG00BEL	88.663	85.303	71.293
WAVR00BEL	21.797	151.829	85.286

**Table 8:** Results in terms of station operation continuity in the Ile-de-France testbed.

KPI	MEL200FRA	MLV200FRA	STC200FRA	VIT200FRA
Number of gaps > 120 s	19	19	18	47
Average length of gaps [s]	108	84	95	125
Max length of gaps [s]	1710	569	1350	2520
Minimum battery voltage [V]	12.58	12.32	12.23	12.34
Average cell signal strength [dBm]	−57.83	−53.42	−64.58	−62.35
Minimum cell signal strength [dBm]	−72.30	−59.30	−77.90	−78.30

**Table 9:** Repeatability of the displacements of the GREAT stations installed in the Ile-de-France region.

KPI	MEL200FRA	MLV200FRA	STC200FRA	VIT200FRA
Number of daily solutions	20	20	20	20
RMS Up displacement [mm]	3.11	2.85	2.79	2.49
RMS East displacement [mm]	0.87	0.69	0.77	0.68
RMS North displacement [mm]	0.62	0.64	0.90	1.00

**Table 10:** Results in terms of station operation continuity in the Wallonia testbed.

KPI	LIEG00BEL	WAVR00BEL
Number of gaps > 120 s	11	8
Average length of gaps [s]	83	79
Max length of gaps [s]	781	990
Minimum battery voltage [V]	11.58	11.57
Average cell signal strength [dBm]	−75.26	−67.85
Minimum cell signal strength [dBm]	−88.10	−88.30

**Table 11:** Repeatability of the displacements of the GREAT stations installed in the Wallonia region.

KPI	LIEG00BEL	WAVR00BEL
Number of daily solutions	20	20
RMS Up displacement [mm]	1.85	1.66
RMS East displacement [mm]	0.57	0.57
RMS North displacement [mm]	0.37	0.65

**Table 12:** Measured power consumption in the different functioning modes.

Operating Mode	Min Power Value MinA × MinV = m_P [W]	Average Power Value (M_P + m_P)/2 = P [W]	Max Power Value MinA × MinV [W] = m_P
GREAT mode with transmission	0.1946 × 12.11 = 2.35	3.06	0.3107 × 12.14 = 3.77
Idle mode with LTE on	0.1894 × 12.12 = 2.29	2.95	0.2974 × 12.14 = 3.61
GREAT mode with no transmission	0.1788 × 12.12 = 2.16	2.39	0.2165 × 12.15 = 2.63
Idle mode no transmission	0.1732 × 12.02 = 2.08	2.28	0.2012 × 12.42 = 2.49

## Data Availability

Results published in this work are based on datasets owned by the GREAT GNSS project consortium and are available upon request.

## Appendix A

This appendix reports in tabular form the achieved results in terms of GNSS data quality assessment in the Ile-de-France and Wallonia testbeds.

**Table A1:** GPS data quality results in the Ile-de-France testbed.

KPI	MEL200FRA		MLV200FRA		STC200FRA		VIT200FRA	
Avg/Min % of acquired epochs	97.0	52.5	97.7	84.2	97.4	62.5	96.5	30.0
Avg/Min % of acquired L1 obs.	80.3	40.7	75.6	46.4	66.9	42.6	80.0	24.9
Avg/Min % of acquired L2 obs.	78.8	38.6	74.2	33.9	65.0	39.5	78.3	20.9
Avg/Min % of acquired L5 obs.	47.4	21.3	49.3	18.8	41.6	21.3	47.4	20.5
Avg/Max % of obs. with cycle slip L1	0.03	0.36	0.02	0.26	0.17	0.72	0.04	0.44
Avg/Max % of obs. with cycle slip L2	0.03	0.36	0.02	0.35	0.15	0.53	0.04	0.57
Avg/Max % of obs. with cycle slip L5	0.03	0.36	0.02	0.27	0.12	0.73	0.03	0.40
Avg/Min C/N0 L1 [dBHz]	44.9	40.0	46.1	41.2	44.3	35.9	45.0	39.3
Avg/Min C/N0 L2 [dBHz]	40.7	36.0	43.3	39.5	39.6	33.3	40.7	35.2
Avg/Min C/N0 L5 [dBHz]	48.0	43.7	49.2	46.0	48.0	41.5	49.0	45.4
Avg/Max multipath L1 [cm]	22.2	28.4	19.3	25.2	17.4	26.3	22.3	31.7
Avg/Max multipath L2 [cm]	10.5	20.7	9.9	18.4	21.6	34.0	15.2	22.9
Avg/Max multipath L5 [cm]	10.5	22.1	7.6	14.2	16.5	42.3	10.1	18.0

**Table A2:** GLONASS data quality results in the Ile-de-France testbed.

KPI	MEL200FRA		MLV200FRA		STC200FRA		VIT200FRA	
Avg/Min % of acquired epochs	97.0	52.5	97.7	84.2	97.4	62.5	96.5	30.0
Avg/Min % of acquired G1 obs.	78.0	26.0	68.2	18.6	57.9	27.1	73.4	31.6
Avg/Min % of acquired G2 obs.	66.8	24.8	57.6	14.9	47.9	23.3	61.5	23.2
Avg/Max % of obs. with cycle slip G1	1.96	10.09	7.66	17.51	1.30	6.60	1.75	12.72
Avg/Max % of obs. with cycle slip G2	0.10	0.75	1.43	16.14	0.42	4.39	0.07	0.54
Avg/Min C/N0 G1 [dBHz]	47.6	43.9	48.7	44.6	46.2	39.8	47.6	44.3
Avg/Min C/N0 G2 [dBHz]	46.4	43.2	48.3	45.0	45.8	32.5	46.7	43.8
Avg/Max multipath G1 [cm]	24.6	35.0	17.5	36.6	34.3	69.4	25.6	39.7
Avg/Max multipath G2 [cm]	12.9	22.1	9.2	19.2	24.6	91.6	14.3	21.2

**Table A3:** GALILEO data quality results in the Ile-de-France testbed.

KPI	MEL200FRA		MLV200FRA		STC200FRA		VIT200FRA	
Avg/Min % of acquired epochs	97.0	52.5	97.7	84.2	97.4	62.5	96.5	30.0
Avg/Min % of acquired E1 obs.	79.6	49.0	65.0	16.1	69.5	21.8	79.1	46.9
Avg/Min % of acquired E5b obs.	79.7	49.0	65.0	16.1	71.8	23.7	79.4	46.9
Avg/Min % of acquired E5a obs.	79.7	49.0	65.0	16.1	71.5	23.3	79.4	46.9
Avg/Max % of obs. with cycle slip E1	0.03	0.34	0.02	0.25	0.18	1.73	0.04	0.42
Avg/Max % of obs. with cycle slip E5b	0.03	0.34	0.02	0.25	0.10	1.07	0.03	0.42
Avg/Max % of obs. with cycle slip E5a	0.03	0.43	0.02	0.26	0.14	1.35	0.03	0.42
Avg/Min C/N0 E1 [dBHz]	43.9	41.8	45.0	42.4	43.8	39.6	44.0	41.6
Avg/Min C/N0 E5b [dBHz]	47.0	44.5	49.0	46.9	47.1	41.3	47.5	44.6
Avg/Min C/N0 E5a [dBHz]	45.4	41.6	46.6	43.2	45.8	39.3	46.6	44.0
Avg/Max multipath E1 [cm]	17.3	23.6	12.7	23.6	12.9	28.0	17.9	26.5
Avg/Max multipath E5b [cm]	9.5	16.0	6.6	13.9	14.1	32.2	8.8	14.2
Avg/Max multipath E5a [cm]	8.6	15.5	6.6	11.2	13.1	31.1	8.3	14.0

**Table A4:** GPS data quality results in the Wallonia testbed.

KPI	LIEG00BEL		WAVR00BEL	
Avg/Min % of acquired epochs	97.7	78.3	97.8	72.5
Avg/Min % of acquired L1 obs.	73.4	55.6	83.7	63.8
Avg/Min % of acquired L2 obs.	73.4	55.6	83.7	63.8
Avg/Min % of acquired L5 obs.	43.3	24.1	49.4	33.4
Avg/Max % of obs. with cycle slip L1	0.02	0.15	0.04	0.44
Avg/Max % of obs. with cycle slip L2	0.02	0.15	0.04	0.44
Avg/Max % of obs. with cycle slip L5	0.02	0.15	0.03	0.31
Avg/Min C/N0 L1 [dBHz]	45.3	43.7	44.2	42.3
Avg/Min C/N0 L2 [dBHz]	41.5	36.9	39.5	34.2
Avg/Min C/N0 L5 [dBHz]	48.0	45.9	47.4	44.6
Avg/Max multipath L1 [cm]	19.6	26.3	22.4	37.4
Avg/Max multipath L2 [cm]	8.9	14.0	13.9	22.0
Avg/Max multipath L5 [cm]	8.8	20.2	11.1	21.4

**Table A5:** GLONASS data quality results in the Wallonia testbed.

KPI	LIEG00BEL		WAVR00BEL	
Avg/Min % of acquired epochs	97.7	78.3	97.8	72.5
Avg/Min % of acquired G1 obs.	72.6	38.7	73.4	40.0
Avg/Min % of acquired G2 obs.	60.9	30.5	61.9	27.1
Avg/Max % of obs. with cycle slip G1	3.15	8.68	1.05	3.65
Avg/Max % of obs. with cycle slip G2	0.06	0.20	0.06	0.34
Avg/Min C/N0 G1 [dBHz]	47.9	45.2	47.1	42.1
Avg/Min C/N0 G2 [dBHz]	47.3	43.9	45.9	39.9
Avg/Max multipath G1 [cm]	17.9	28.4	23.9	41.2
Avg/Max multipath G2 [cm]	10.3	18.1	15.7	31.2

**Table A6:** GALILEO data quality results in the Wallonia testbed.

KPI	LIEG00BEL		WAVR00BEL	
Avg/Min % of acquired epochs	97.7	78.3	97.8	72.5
Avg/Min % of acquired E1 obs.	74.1	48.2	85.2	66.0
Avg/Min % of acquired E5b obs.	74.1	48.3	85.5	66.0
Avg/Min % of acquired E5a obs.	74.1	48.3	85.5	66.1
Avg/Max % of obs. with cycle slip E1	0.02	0.14	0.06	0.57
Avg/Max % of obs. with cycle slip E5b	0.02	0.14	0.03	0.29
Avg/Max % of obs. with cycle slip E5a	0.02	0.14	0.03	0.29
Avg/Min C/N0 E1 [dBHz]	43.9	41.3	42.8	40.2
Avg/Min C/N0 E5b [dBHz]	47.8	45.4	46.6	43.4
Avg/Min C/N0 E5a [dBHz]	45.6	43.0	44.9	42.1
Avg/Max multipath E1 [cm]	14.5	23.6	19.6	33.8
Avg/Max multipath E5b [cm]	7.4	11.7	10.6	17.6
Avg/Max multipath E5a [cm]	8.1	14.8	10.0	17.6
